# Fiber Bragg Grating Temperature Sensors in a 6.5-MW Generator Exciter Bridge and the Development and Simulation of Its Thermal Model

**DOI:** 10.3390/s140916651

**Published:** 2014-09-05

**Authors:** Kleiton de Morais Sousa, Werner Probst, Fernando Bortolotti, Cicero Martelli, Jean Carlos Cardozo da Silva

**Affiliations:** 1 Graduate Program in Electrical and Computer Engineering, Federal University of Technology-Paraná, Curitiba-PR 80230-901, Brazil; E-Mails: kleitonsousa@utfpr.edu.br (K.M.S.); cmartelli@utfpr.edu.br (C.M.); 2 University of Applied Science, Munich 80335, Germany; E-Mail: werner.probst@bayern-mail.de; 3 Electrical Engineering Graduate Program, Federal University of Technology-Paraná, Pato Branco-PR 85503-390, Brazil; E-Mail: fernando.bortolotti@copel.com

**Keywords:** fiber Bragg grating, generator excitation bridge, semiconductor thermal model, temperature measurement, thermal model simulation

## Abstract

This work reports the thermal modeling and characterization of a thyristor. The thyristor is used in a 6.5-MW generator excitation bridge. Temperature measurements are performed using fiber Bragg grating (FBG) sensors. These sensors have the benefits of being totally passive and immune to electromagnetic interference and also multiplexed in a single fiber. The thyristor thermal model consists of a second order equivalent electric circuit, and its power losses lead to an increase in temperature, while the losses are calculated on the basis of the excitation current in the generator. Six multiplexed FBGs are used to measure temperature and are embedded to avoid the effect of the strain sensitivity. The presented results show a relationship between field current and temperature oscillation and prove that this current can be used to determine the thermal model of a thyristor. The thermal model simulation presents an error of 1.5 °C, while the FBG used allows for the determination of the thermal behavior and the field current dependence. Since the temperature is a function of the field current, the corresponding simulation can be used to estimate the temperature in the thyristors.

## 1. Introduction

One of the most crucial issues of package technology for power semiconductor devices is the thermal performance. According to the literature [1], nearly 60% of failures are temperature-induced, and for every increase of 10 °C in the temperature of the operating environment, the failure rate nearly doubles. Power plants have thermal protectors for all parts of a generator. However, the exciter bridge does not have any thermal measurement or protection. Furthermore, the generator excitation current is adjusted to the reactive power. Due to this characteristic, the excitation bridge thermal behavior is a function of excitation current and can also be related to the active power. Besides, temperature measurement in the excitation bridge can be used to determine faults in the generator rotor.

To characterize the dynamic thermal behavior, semiconductor manufacturers typically provide the thermal impedance curve. This curve shows the transient behavior of the peak temperature for a step application of a constant power. In many cases, it is necessary to accurately predict the thermal behavior in the microsecond time scale, which complicates the experimental measurements [2,3]. For this application, some benefits over traditional electrical sensors are demonstrated. The fiber Bragg gratings (FBGs) are totally passive, being immune to electromagnetic interference and capable of being multiplexed in a single fiber. With respect to their dimensions, they are smaller than their most used electrical counterparts. The FBG has a fast response, in order of the semiconductor temperature transient, due to its small mass [4]. The use of FBG sensors was previously reported in [3], where an IGBTthermal model was presented.

Due to such characteristics, the FBG has a great potential for use in energy conversion systems. In [5], the temperature behavior of a small single-phase induction motor is presented using FBG, and in [6], the FBG was used to determine the induction motor thermal model. In [7], the FBGs were used for the measurement of temperature in electric power generators in order to replace the PT 100 sensors in an experimental generator. Another FBG application in an electric power generator is presented in [8], where six multiplex FBGs are used for stator temperature measurement.

**Figure 1.**
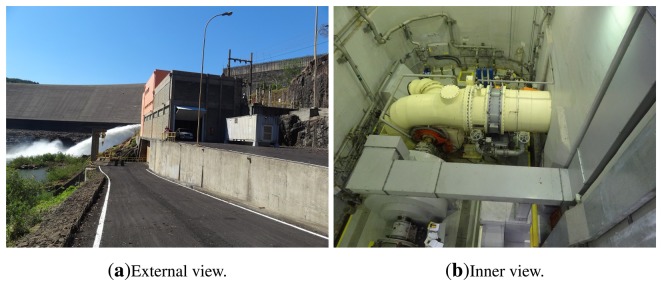
View of the hydroelectric power plant. The power plant has a 6.5-MW power generator.

This paper presents a thermal measurement and a thermal model simulation of a 6.5-MW generator exciter bridge. The exciter bridge is formed by six by six thyristors. For the temperature measurement, six FBG are used. The measurements are performed in a hydroelectric power plant at Jordão River, in the Brazilian state of Paraná. The owner of this plant is COPEL, Paraná's state power utility company. Figure 1 shows a view of the hydroelectric power plant and powerhouse.

The studied power generator has a static three-phase-bridge thyristor rectifier system to control and maintain the generator reactive power at predetermined values over the output voltage. The core of the excitation system is a three-phase-rectifier-bridge, which has two functions: rectifying the alternating voltage into direct voltage and controlling the field current flow through the rotor, namely the excitation. As already stated, the field current produces the magnetic field over the field coil. Having a constant rotation of the shaft, the magnetic flow determines the voltage induced inside the stator. On the other side, the induced voltage directly influences the output voltage of the generator, which is the voltage provided to the grid after rectification. By controlling the rotor excitation, the output values of the system, the voltage, and the reactive power are controlled at the same time.

The instrumentation of the bridge rectifier is important due to the fact that this device usually does not measure the temperature in hydroelectric power plants. The monitoring of the temperature can prevent failures due to overheating of the thyristors, and their thermal model can be used to estimate the temperature of the thyristors without using sensors. The temperature estimation is a function of the field current, and this is the input parameter for the thermal model. As a consequence, the thyristor temperature can be determined by measuring the field current and using the thermal model. Furthermore, as the temperature of the thyristors depends on the field current, a measured or simulated temperature can be used to determine the conditions of the generator operation.

## 2. Experimental Setup

The fiber Bragg gratings (FBG) used in this paper were imprinted inside the core of optical fibers by using the phase-mask technique. The procedure was performed in the Photo-Refractive Devices Unit at the Federal University of Technology, Paraná. The set of sensors is formed by six wavelength-multiplexed FBGs in a range of 1532 nm up to 1551 nm and with Bragg wavelengths at every 4 nm. The FBGs were recorded in a standard single mode optical fiber using the phase mask technique and a excimer laser at 193 nm, and and with this setup, a reflectivity of 70% was obtained. The fibers are not in annealed form for use in this work.

In order to reduce the intrinsic FBG sensitivity to strain, which would distort the temperature readings, an encapsulation of the sensor was elaborated. Furthermore, this encapsulation makes the FBG sensor robust for industrial applications. This is worth noticing, because the fiber region where the fiber section on which the FBG is imprinted turns out to be very fragile, due to the removal of the acrylate and the exposure of the fiber to a UV laser during its fabrication process. The encapsulation consists of a steel tube with dimensions of 0.6 × 25 mm, and its ends are fixed with epoxy glue. The usage of this glue is based on the fact that it tolerates temperatures up 100 °C, being pliable and able to facilitate the positioning of the sensors in the heat sink.

**Figure 2.**
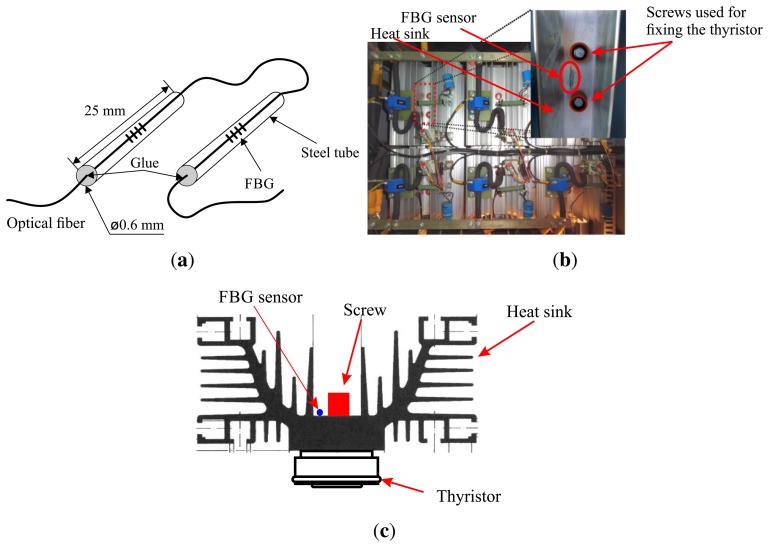
**(a)** The developed encapsulation to fix the fiber Bragg grating (FBG) in the heat sink; and **(b)** photograph of the rectifier bridge with the heat sink and a close-up view of the mounted sensor placed in the back side of the heat sink; **(c)** Cross view of the heat sink.

The illustration of the FBG encapsulation is shown in Figure 2a, presenting the dimensions of the steel tube and the fastening points with glue. The six FBGs are temperature characterized using a circulating bath with a digital temperature control model Lauda Eco Gold RE 415G with a precision of 0.02 °C and a stabilization error of 0.01 °C. The calibration of the sensors is realized for 15 samples and shows repeatability and precision in the measurement process, with a standard deviation of less than 4 pm. The calibration of the sensors was performed using the FBG encapsulated FBG and, therefore, incorporating the effects of the glue and steel tube in the measurements. After the calibration of the sensors, the uncertainties can be obtained, and the confidence intervals for the temperature measurements are calculated. The temperature coefficient of the sensors after calibration is 0.013 pm/°C. The FBG has a typical temperature coefficient of 0.010 nm/°C, so the value of 0.013 pm/°C is obtained, taking into account the effect of the encapsulation. With the standard deviation value and optical interrogator precision, expanded uncertainties of 0.47 °C are obtained [9].

**Figure 3.**
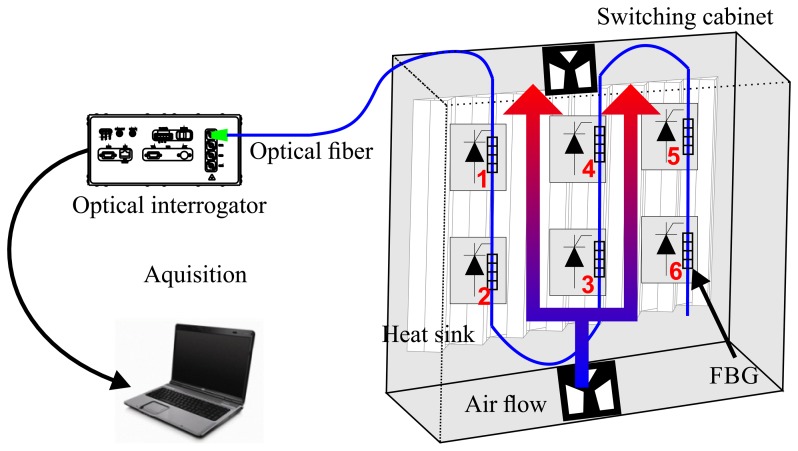
Schematic view of the experimental assembly: switching cabinet with FBG sensors above the rectifier thyristors; the ventilation system is depicted with the air flow illustration; the numbers of the sensors are in red; the optical interrogator and laptop are depicted as the monitoring device.

The optical fiber cable containing the six FBG sensors is installed on the heat sink directly above the thyristors, as shown in Figure 2b. In the switching cabinet, the thyristors are fixed on the heat sink using screws and cannot be viewed in Figure 2b. The Figure 2c shows a cross view of the heat sink, where the position of the thyristor can be observed. Fastening was performed with epoxy-based glue, which is resistant up to 100 °C, reinforcing thermal conductivity and providing a mechanical bond between the heat sink and the sensors. A schematic drawing of the assembly is shown in Figure 3, where the numbers in red indicate the position of the FBG sensors. The optical link is connected with the HBM's DI-410 interrogator.

## 3. Thyristor Temperature Measurement

**Figure 4.**
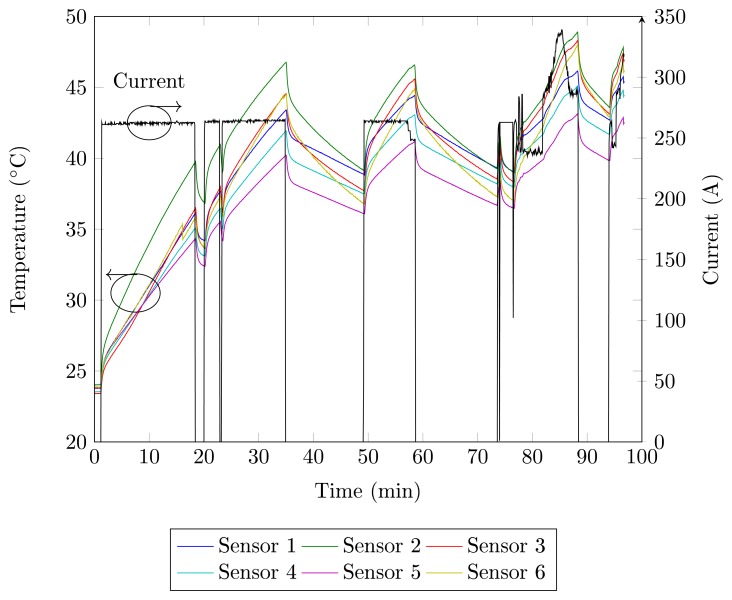
Temperature behavior measured by the six FBG sensors and the current behavior (black) as functions of time.

Tests are performed in order to determine the temperature behavior of thyristors and the relationship with the field current. The measurement results for the six FBG sensors are presented in Figure 4. An initial glance at the graph in Figure 4 indicates the similar behavior between all temperature measurements during startup, differing mostly in the thermal amplitude reached, which results in differences in the slope of the curve. The temperature increase is caused by the power losses, while the current is conducted by the thyristor.

The heat transfer occurs in the direction of the switching cabinet, which is a colder region, as sketched in Figure 3, and the three different types of heat transfer are evaluated. Heat convection transfers heat through a cooling flow from the bottom, where air enters through a filter to the top, leaving the cabinet, due to air aspirating fans. The air heating up while crossing the components to the top and the thyristors positioned at the lower row show lower temperatures as those in the upper row. The radiation of heat emitting components happens in the direction of the surface. Through the heat sink, heat conduction arrives the same conclusion: as the temperature differences between the heat sink and the components decrease from the outside to the middle, less heat from a thyristor in the middle of a switching cabinet can be transferred.

**Figure 5.**
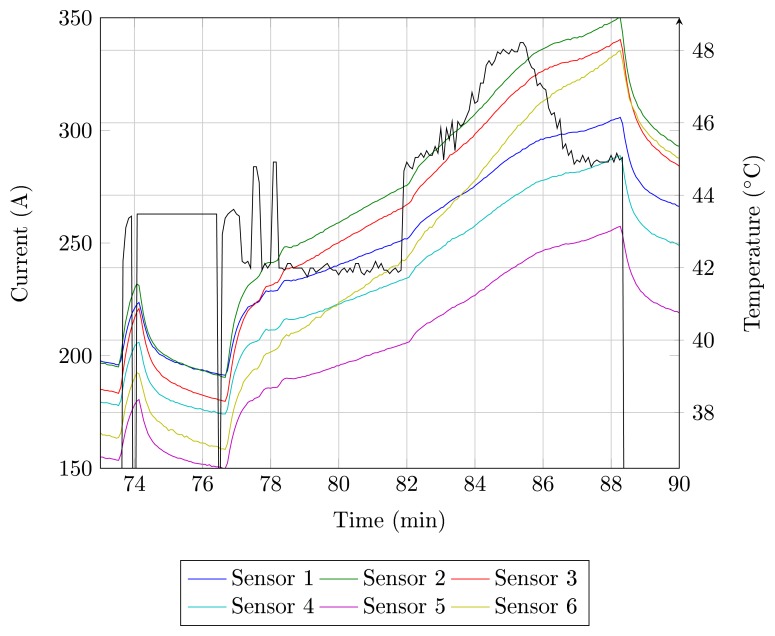
Temperature measurement detail of the six FBG sensors.

In Figure 4, the generator synchronization to the grid occurs at *t* = 80 min. The current variation occurs to adjust the reactive power provided by the generator. During the current variation for *t* > 80 min, the sensors temperature shows the same current profile. Figure 5 shows the current and temperature detail for 74 > *t* > 90 min. In this figure, it can be observed that there is a relation between the field current and the temperature of the thyristors. The current oscillations cause temperature fluctuations, as well as a current step change causes the temperature to rise to a higher level. These results show that the field current can be used to determine the thermal model of the thyristor. According to the manufacturer, the maximum thyristor temperature is 125 °C, but in the operation regime, the maximum temperature is 86 °C. During the tests, the temperature of the thyristors remained below these values.

## 4. Thyristor Thermal Model

### 4.1. Parameters of Thyristor Thermal Model

**Figure 6.**
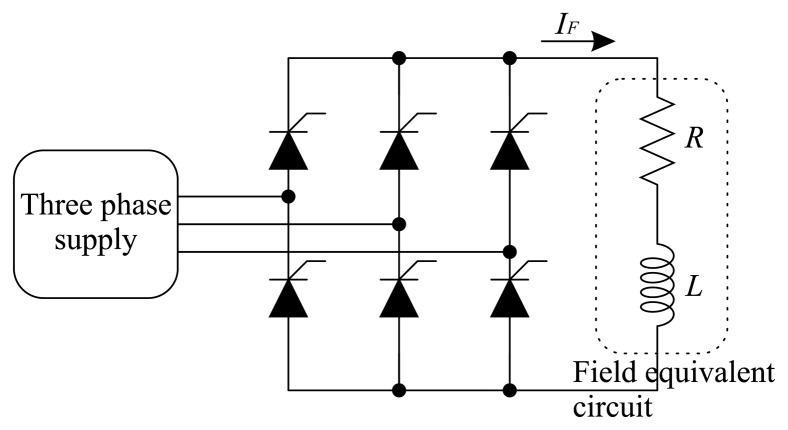
Generator excitation equivalent circuit. The circuit has an *RL* series load and a field current *I_F_*.

The electric generator excitation has the function of regulating the output generator voltage. The generator excitation circuit consists of a three-phase Graetz bridge. The three-phase supply is derived from the generator itself, in the case of a self-excited system. When the generator is switched off, the residual magnetism of the rotor ensures its re-start-up when it operates again. Furthermore, batteries are used to power the excitation for a short period of time, with no influence on the temperature of the thyristors. The rectifier load is modeled by an *RL* series branch, representing the rotor windings. Figure 6 shows the circuit of the generator excitation.

**Figure 7.**
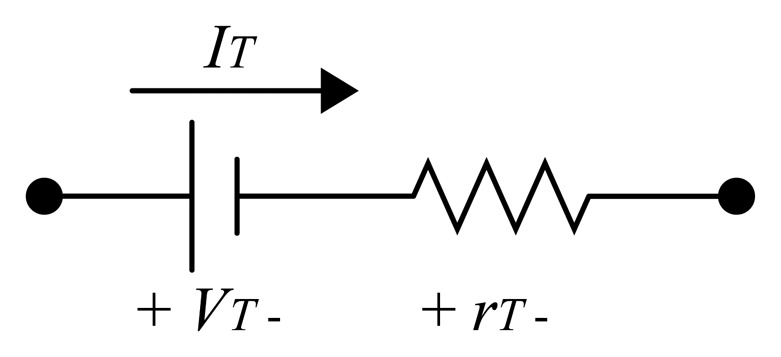
Thyristor equivalent circuit in conduction mode. In conduction mode, the thyristor is modeled by a voltage source *V_t_* in series with a resistance *r_T_*; the thyristor current is represented by *I_T_*.

The losses in the semiconductor devices lead to an increase in the temperature. These losses are divided into switching losses and conduction losses. Thyristor switching occurs at low frequencies, usually at 50 Hz and 60 Hz. For this reason, the thyristor switching losses are neglected. The conduction losses are calculated from the thyristor equivalent circuit, shown in Figure 7. The model of a thyristor in conduction mode has a voltage drop *V_T_* and a resistance *r_T_*. Thus, the thyristor losses are obtained from the power in *V_T_* and *r_T_*, where the thyristor current *I_T_* is conducted. The losses are calculated by:

(1) $$P=I_{\text{Tavg}}V_{T}+I_{\text{Trms}}^{2}r_{T}$$

where *P* is the power losses, *I_Tavg_* is the average thyristor current and the *I_Trms_* is the root-mean squared (RMS) thyristor current.

The generator excitation has an inductance that is large enough to ensure that the current *I_F_* is continuous and has no oscillation, regardless of the changes in the voltage field. Thus, one can obtain a simplified expression for the current *I_T_* as a function of *I_F_*. In each phase rectifier, the thyristor conducts the current during 1/3 of the period. Therefore, the thyristor average and RMS currents are given by:

(2) $$I_{\text{Tavg}}=\frac{I_{F}}{3}$$

(3) $$I_{\text{Trms}}=\frac{I_{F}}{\sqrt{3}}$$

Thus, the thyristor losses equation is written as a function of field current *I_F_*:

(4) $$P=\frac{I_{F}}{3}V_{T}+\frac{I_{F}^{2}}{3}r_{T}$$

the thyristor losses are the input of the equivalent thermal circuit. The datasheets of the semiconductor devices contain the curves with values of *V_T_* and *r_T_*. These parameters are not constant and are a function of the thyristor current *I_T_*.

**Figure 8.**
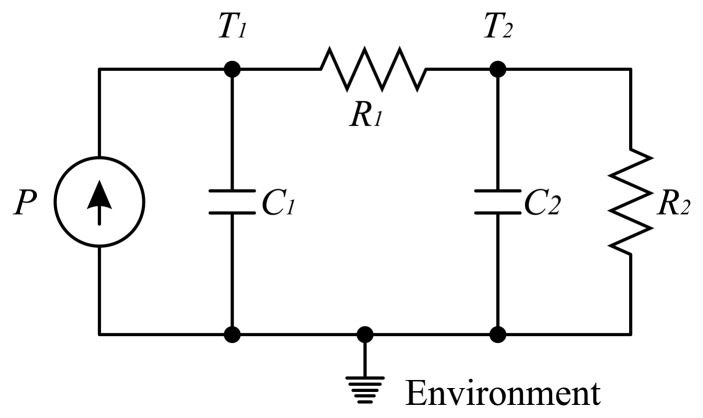
Equivalent thermal circuit for the thyristor and heat sink. The thermal resistance between the capsule and the heat sink is represented by *R*_1_. C_1_ represents the thyristor thermal capacitance and *C*_2_ represents the heat sink thermal capacitance. The thermal resistance between the heat sink and the environment is represented by *R*_2_.

The thyristor thermal model can be represented by an equivalent thermal circuit, composed of thermal resistances and capacitances [3,10-12]. The thermal circuit is shown in Figure 8. Candidate models are often comprised of more complex mathematical functions, which do not offer such intuitive insight. Such models can be stated in frequency or time domains and are not generally amicable to simple physical interpretation [11]. A good approximation for the thyristor thermal circuit is a second order system. Other simulations of the model used a finite-difference thermal model or Fourier series solutions of a heat equation [13]. However, both models lead to either complicated analytical expressions or numerical-only results [14]. Therefore, it is not possible to determine the parameters for these models, since the thyristors used in this paper are in an industrial environment.

The power losses lead to an increase in the temperature at the junction of the thyristor. The junction is in direct contact with the thyristor capsule. The thyristor capsule is in contact with the environment and has a certain thermal capacitance and resistance between the junction and the capsule. The capsule also has a thermal capacitance and resistance between the capsule and the environment. Thermal resistances are informed by the thyristor and the heat sink manufacturers. Thermal capacitances are obtained experimentally, using the transient temperature of the thyristor.

However, the thyristors are positioned on a heat sink, leading to an increase in the system order. Since the heat sink has a higher time constant compared to the time constant of the junction and capsule, it is necessary to reduce the system order, where the junction and the capsule provide only an equivalent time constant. This simplification is already considered in the thermal circuit of Figure 8, where *C*_1_ is the thermal capacitance of the junction-capsule and *R*_1_ is the thermal resistance between the capsule and the heat sink. The heat sink has a thermal capacitance *C*_2_, and *R*_2_ is the thermal resistance between the heat sink and the environment. Still in Figure 8, *T*_1_ and *T*_2_ are the temperatures of the capsule and the heat sink, respectively.

The thermal model state space equation is given by Equation (5):

(5) $$\left[\begin{array}{l} T_{1}\\ T_{2} \end{array}\right]=\left[\begin{array}{cc} -\frac{1}{R_{1}C_{1}} & \frac{1}{R_{1}C_{1}}\\ \frac{1}{R_{1}C_{2}} & -\frac{1}{C_{2}}\left(\frac{1}{R_{1}}+\frac{1}{R_{2}}\right) \end{array}\right]\;\left[\begin{array}{l} T_{1}\\ T_{2} \end{array}\right]+\left[\begin{array}{c} \frac{1}{C_{1}}\\ 0 \end{array}\right]P$$

and the measurement is performed in the heat sink, so it is important to determine the system's transfer function. This transfer function is given by:

(6) $$\frac{T_{2}(s)}{P(s)}=\frac{R_{2}}{s^{2}C_{1}C_{2}R_{1}R_{2}+s[R_{1}C_{2}+R_{2}(C_{1}+C_{2})]+1}$$

where *s* is a complex variable in the Laplace transform.

The system step response is analyzed in order to determine the parameters of the system in Figure 8. The analysis of the transient dynamics in the electrical and mechanical systems frequently utilizes the step-response technique. Here, an instantaneous step-change in system inputs or disturbances is used to reveal the behavior of the physical system. The transition between pre-step and post-step equilibrium conditions contains all of the information needed to synthesize a dynamic model capable of simulating or predicting the response of the linear physical system to any disturbance or operating condition [11].

### 4.2. Simulation of the Thyristor Thermal Model

**Figure 9.**
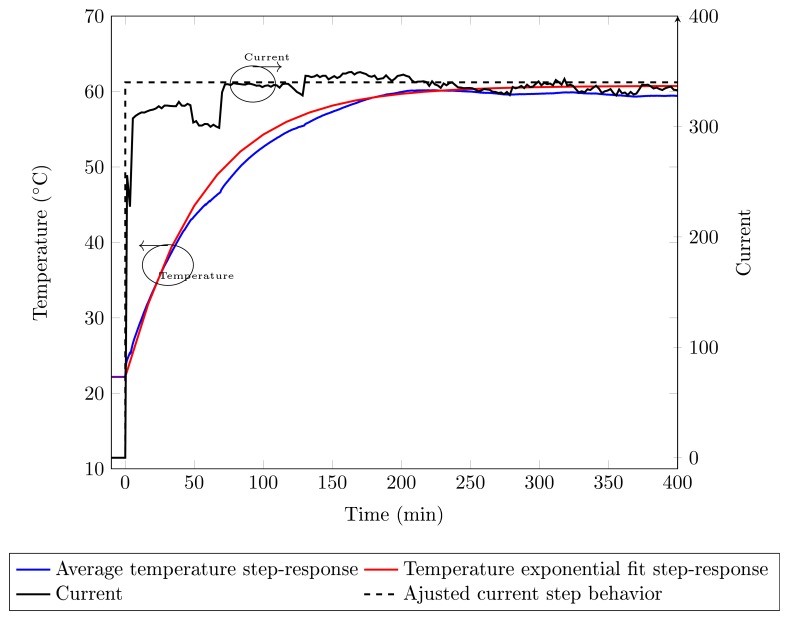
On the left axis, the corresponding axis to the average temperature in the six FBG sensors' (blue) curves and the exponential curve (red); on the right, the corresponding current (solid black) and the adjusted step current (dashed black) curves.

The analysis of step-response dynamics is used to determine the parameters of the model shown in Figure 8. A step current is applied to the circuit excitation while the generator is connected to the grid. The average measured temperature step-response is presented in Figure 9 (solid blue line). Due to the fact that the generator is connected to the grid, some current adjustments are necessary in order to maintain the correct generator operation; this would also lead to oscillations in the current, as depicted in Figure 9 (solid black line). However, the current presents a similar step behavior. Therefore, in order to determine the thyristor thermal model, it is necessary to approximate the field current by a current with a step behavior. The current step behavior approximation is depicted in Figure 9 (black dashed line), and the current value is *I_F_* = 365 A. A second order exponential function is used to fit the average temperature, having the same order of the thermal model in Figure 8, depicted in Figure 9.

The current field is used to calculate the thyristor losses. For *I_F_* = 365 A, the thyristor average current is *I_T_* = 121.67 A, according to (2). The parameters *V_T_* and *r_T_* are obtained by the thyristor datasheet and *V_T_* = 0.9 V and *r_T_* = 0.38 mΩ for *I_T_* = 121.67 A. The values of *V_T_* and *r_T_* are not constant and are a function of the thyristor current. However, the values of *V_T_* and *r_T_* are constant for a step current. The thyristor losses calculated by (4) are *P* = 126.4 W for *I_F_* = 365 A.

The temperature step response presented in Figure 9 is fit by a second order exponential and is given by:

(7) $$\Delta T(t)=38.58+0.4802e^{-0.00244t}-39.0595e^{-0.0003t}$$

and the transfer function, considering a step, is given by:

(8) $$\frac{\Delta T(s)}{P(s)}=\frac{0.0002824}{s^{2}+0.02471s+7.728^{-6}}$$

The parameters *R*_1_ and *R*_2_ are obtained from the thyristor and heat sink datasheets, and *R*_1_ = 0.04 °C/W and *R*_2_ = 0.29 °C/W. Thermal capacitances are obtained comparing the Expressions (6) and (8). The capacitances are *C*_1_ = 1500 J/°C and *C*_2_ = 8500 J/°C.

**Figure 10.**
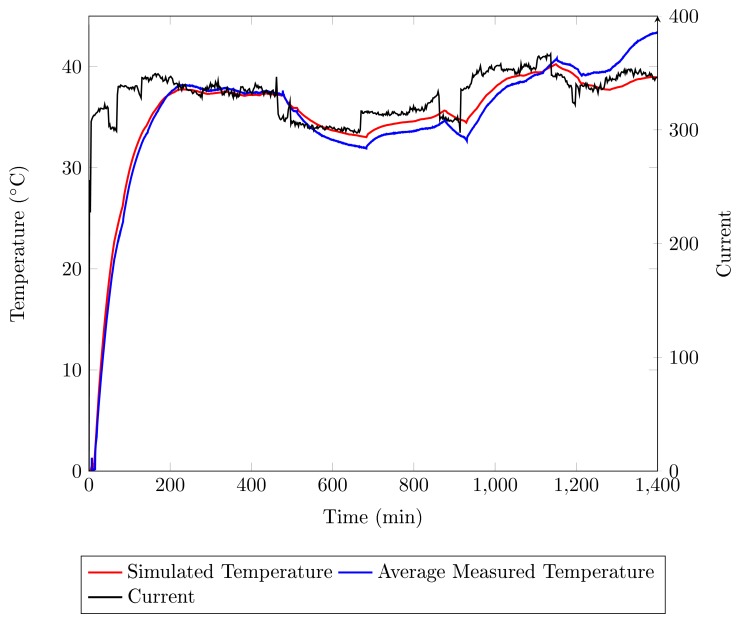
Temperature and current measurement during 24 h. On the left, the axis corresponding to the average temperature curves in the six FBG sensors (blue line) and the simulated temperature using the thermal model (red line); on the right, the current axis corresponding to the current curve (black) as a function of time.

The temperature simulation during 24 h of the generator operation is presented in Figure 10. The state-space equation in (5) is simulated using the Euler numeric method with a step simulation time of 5 s. Simulated temperature (red line) and average temperature (blue line) present a similar behavior as a function of field current. From the simulation results, it can be confirmed that the thermal model used, as well as the thermal parameters present a good approximation when comparing the average temperature. The maximum difference between the simulated and measured temperatures during the transient is 0.5 °C.

From 0 to 500 min, the simulated and average temperatures present similar behavior. After that, for 500 < *t* < 1100 min, the average temperature is 1.5 °C less than the simulated temperature. This occurs due to a drop in room temperature during the night. For *t* > 1100 min, during the day, the room temperature increases, and the average temperature is greater than the simulated temperature. The simulation does not take the room temperature into account.

There are errors when the simulation is compared with the sensors individually. These errors can be attributed to factors that are not taken into account in the model presented in the paper, as in the case of the construction differences in the semiconductors: unbalanced current in the thyristors, air flow in the cabinet and constructive differences in the heat sinks. Because this is an installation in an industrial environment, it is not possible to perform other tests to determine these parameters or a more detailed model.

## 5. Conclusions

In this paper, the use of FBG sensors in a 6.5-MW generator rectifier bridge allows the determination of its thermal behavior and the field current dependence. It has been determined that a variation in the field current leads to changes in the temperature, and the temperature monitoring process is used to determine a second order thermal model. In addition, the thermal model parameters are determined from a current step response and using the component's datasheet. The thermal model simulation presents an error of 1.5 °C in comparison with the average temperature. The differences between the simulated temperature and the average temperature are due to changes in ambient temperature. These temperature changes are not taken into account in the model simulation.

Temperature simulation can be used to estimate the temperature in the thyristors. Moreover, since the thyristor temperature is a function of the field current, the generator operation condition can be determined. In the case of the field current, the generator reactive power can be determined using the knowledge of its temperature. On the other hand, the thyristor temperature measurement can be used to determine the rotor generator condition from an indirect measurement.
